# Charakterisierung der Patienten mit Synkope in der Notaufnahme – Nebendiagnosen und Laborparameter bei stationärer und ambulanter Versorgung

**DOI:** 10.1007/s00063-024-01241-w

**Published:** 2025-02-07

**Authors:** Aaron Becker von Rose, Adrian Patenge, Bernhard Haller, Niel Mehraein, Lisa Schmid, Dominik Pförringer, Michael Dommasch

**Affiliations:** 1 TUM School of Medicine and Health, Zentrale Interdisziplinäre Notaufnahme, TUM-Universitätsklinikum, Technische Universität München, Ismaninger Str. 22, 81675 München, Deutschland; 2 TUM School of Medicine and Health, Klinik und Poliklinik für Unfallchirurgie, TUM-Universitätsklinikum, Technische Universität München, Ismaninger Str. 22, 81675 München, Deutschland; 3 Institut für KI und Informatik in der Medizin, TUM-Universitätsklinikum, Technische Universität München, Ismaninger Str. 22, 81675 München, Deutschland

**Keywords:** Reflexsynkope, Vasovagale Synkope (VVS), Orthostatische Synkope, Arrhythmogene Synkope, Koronare Herzkrankheit, Transient, Loss of Consciousness, Reflex syncope, Vasovagal syncope, Cardiac syncope, Orthostatic syncope

## Abstract

Insgesamt 1391 Patienten stellten sich in den Jahren 2019–2022 in der Notaufnahme des Klinikums rechts der Isar der Technischen Universität München mit der Hauptdiagnose Synkope vor. In dieser Studie wurden diejenigen Patienten analysiert, die sich in einer interdisziplinären Notaufnahme vorgestellt haben. Patienten, die über den Schockraum oder über die Chest Pain Unit aufgenommen wurden, sind in diesem Kollektiv nicht enthalten. Ziel dieser monozentrischen retrospektiven Studie ist die Charakterisierung im Hinblick auf Nebendiagnosen und Laborparametern. Besonderer Fokus lag hierbei auf der Differenzierung zwischen stationären (*n* = 190; 13,7 %) und ambulanten (*n* = 1201; 86,3 %) Patienten. Die häufigsten Nebendiagnosen waren Kopfverletzung (*n* = 188; 13,5 %), Infektion (*n* = 126; 9,1 %), sonstige Verletzung des Körpers (*n* = 124; 8,9 %), neurologische Erkrankung (*n* = 85; 6,1 %), rhythmogene Herzinsuffizienz (*n* = 76; 5,5 %), kardiovaskuläre Risikofaktoren (*n* = 75; 5,4 %), metabolische/nephrologische Erkrankung (*n* = 69; 5,0 %) und strukturelle Herzerkrankungen (*n* = 68; 4,9 %). Operative Interventionen erfolgten mittels Herzkatheter (*n* = 16; 1,2 %), perkutaner Koronarintervention (*n* = 15; 1,1 %) und Schrittmacher/Defibrillator/Ereignisrekorder (*n* = 12; 0,9 %). Patienten, die stationär versorgt wurden, wiesen im Vergleich zu ambulanten Patienten im Durchschnitt deutlich mehr Nebendiagnosen (3,5 vs. 0,3) und höhere Inzidenzen pathologischer Laborparameter auf. Häufigere Nebendiagnosen bei stationären Patienten waren kardiovaskuläre Risikofaktoren (37,9 % vs. 0,2 %), rhythmogene Herzinsuffizienz (35,3 % vs. 0,7 %), Infektion (57,9 % vs. 1,3 %) und Lungenerkrankung (6,3 % vs. 0,2 %). Bei stationären Patienten zeigten sich häufig abnormale Blutwerte wie erhöhte Laborparameter für „high sensitive“ (hs) Troponin T (58,8 % vs. 25,7 %), Kreatinin (36,2 % vs. 14,8 %) und Leukozyten (43,4 % vs. 36,3 %) sowie erniedrigte Werte für Hämoglobin (33,3 % vs. 16,0 %), Kalium (5,3 % vs. 1,2 %) und Natrium (2,1 % vs. 0,6 %).

**Zusatzmaterial online:**

Zusätzliche Informationen sind in der Online-Version dieses Artikels (10.1007/s00063-024-01241-w) enthalten.

## Abstract (en)

Over 1391 patients presented to the emergency department (ED) of a German university hospital with primary diagnosis of syncope from 2019–2022. This monocentric, retrospective study aims to characterize this cohort regarding secondary diagnoses and blood laboratory parameters. Principal focus lay on the differentiation between inpatients (*n* = 190; 13.7%) and outpatients (*n* = 1201; 86.3%). Most common secondary diagnoses comprised head injury (*n* = 188; 13.5%), infection (*n* = 126; 9.1%), body injury (*n* = 124; 8.9%), neurological disease (*n* = 85; 6.1%), arrhythmogenic heart failure (*n* = 76; 5.5%), cardiovascular risk factors (*n* = 75; 5.4%), metabolic/nephrological disease (*n* = 69; 5.0%), and structural heart disease (*n* = 68; 4.9%). Surgical interventions were performed using catheter (*n* = 16; 1.2%), percutaneous coronary interventions (*n* = 15; 1.1%), and pacemaker/defibrillator/event recorders (*n* = 12; 0.9%). Inpatients had significantly more secondary diagnoses (3.5 vs. 0.3) and higher incidences of abnormal laboratory parameters compared to outpatients. Secondary diagnoses more common in inpatients included cardiovascular risk factors (37.9 vs. 0.2%), arrhythmogenic heart failure (35.3 vs. 0.7%), infection (57.9 vs. 1.3%), and lung disease (6.3 vs. 0.2%). Abnormal blood laboratory values more frequent in inpatients included elevated levels of highly sensitive (hs) troponin T (58.8 vs. 25.7%), creatinine (36.2 vs. 14.8%), leukocytes (43.4 vs. 36.3%), besides decreased hemoglobin (33.3 vs. 16.0%), potassium (5.3 vs. 1.2%), and sodium (2.1 vs. 0.6%).

## Einleitung

Die Synkope ist das häufigste Symptom bei Patienten mit „transient loss of consciousness“ (TLoC). TLoC ist charakterisiert als temporärer Bewusstseinsverlust mit vollständiger Rückbildung. Als Differenzialdiagnosen sind v. a. epileptische oder psychogene Anfälle und transitorische ischämische Attacken (TIA) von Bedeutung. Synkopen in der Notaufnahme sind häufig und bedürfen einer sorgfältigen Diagnostik, die in der Regel internistisch/kardiologisch und/oder neurologisch erfolgt [1, 2]. Priorität hat hierbei die Abklärung potenziell lebensbedrohlicher Erkrankungen des Herzens. Die initiale Diagnostik umfasst typischerweise die Anamnese und körperliche Untersuchung, ein 12-Kanal-Elektrokardiogramm (EKG), die Blutdruckmessung im Liegen und Stehen sowie eine BGA bzw. ein Blutbild zur weiteren Differenzialdiagnostik (Fokus auf Entzündungswerte sowie Nierenwerte). Die Synkope kann ätiologisch in 3 übergeordnete Kategorien unterteilt werden: Reflex- oder vasovagale Synkope (VVS), Synkope aufgrund orthostatischer Hypotonie (OH) und kardiale Synkope [1, 2]. Bei der kardialen Synkope wird ferner zwischen arrhythmogener Synkope und Synkope aufgrund struktureller Herz- oder Gefäßerkrankung unterschieden. Das durch Flüssigkeitsmangel verursachte posturale Tachykardiesyndrom (POTS) wird nicht als eigenständiger Synkopemechanismus, sondern als Risikofaktor für eine orthostatische VVS angesehen [3]. Das Therapieziel bei Patienten mit Synkope ist primär abhängig von der initialen Diagnostik sowie Risikostratifizierung. Generell stehen die Verhinderung einer erneuten Synkope sowie der Ausschluss eines epileptischen Geschehens als häufigste Differenzialdiagnose im Vordergrund [4]. Von einer Niedrigrisikosynkope ist auszugehen, sobald bei unauffälliger Grundanamnese sowie körperlicher Untersuchung ebenfalls keine Auffälligkeiten in der BGA sowie im EKG zu erkennen sind. Als Ursachen dieser sog. PPP-Synkope (Position, Provokation, Prodromi) gelten schnelle Positionsveränderungen (liegen zu stehen), visuelle Auslöser oder auch Schmerz. Patienten beschreiben zugehöriges „Ohrensausen“ sowie, als wäre es kurzzeitig „schwarz vor Augen“ geworden [1]. Intermediärsynkopen beschreiben etwaige Synkopen unklarer Genese, die ohne eindeutigen Auslöser eine umfangreichere Diagnostik, ggf. auch unter stationären Bedingungen, notwendig machen. Als sog. Hochrisikosynkopen, die immer eine stationäre Aufnahme bedingen, gelten Fälle, die eines der sog. CHESS-Kriterien erfüllen, die in der San-Francisco Syncope Rule (SFSR) aufgeführt sind [5]: Sollte ein betroffener Synkopepatient in der Vorgeschichte eine bekannte Herzerkrankung („congestive heart failure“) aufweisen, der Hämatokrit unter 30 % liegen („hematocrit“) bzw. Abnormalitäten im EKG (ECG) auftreten, eine Dyspnoe („shortness of breath“) pre- bzw. postsynkopal zeigen oder der initial triagierte systolische Blutdruck < 90 mm Hg liegen („systolic blood pressure“), besteht ein hohes Risiko für ein schlechtes Outcome [1, 5]. Neben anderen Kriterien bzw. Risikostratifizierungsmethoden in der Notfallmedizin (siehe [6]), gelten die beschriebenen CHESS-Kriterien als sog. Red Flags für Patienten mit Synkope in der interdisziplinären Notaufnahme. Bewährte Therapien der Synkope richten sich nach der spezifischen Genese und umfassen neben der Vermeidung auslösender Faktoren (beispielsweise Schmerz oder schnelle Positionswechsel) auch interventionelle Maßnahmen. Die Implantation eines Herzschrittmachers oder Defibrillators und die Katheterablation im Kontext der kardialen Synkopen sind beispielhaft als häufigste Interventionen zu nennen. Zur Vermeidung des plötzlichen Herztods als gravierendste Komplikation einer kardialen Synkope ist die stationäre Beobachtung solcher Synkopen obligat. Nichtkardiale Synkopen können hingegen im Regelfall ambulant behandelt werden [1].

## Patienten und Methoden

Die Patientenidentifikation erfolgte durch Abfrage aller Notfallpatienten mit ICD-codierter Hauptdiagnose R55 „Synkope und Kollaps“ innerhalb des Zeitraums von 2019 bis 2022 im Krankenhausinformationssystem (KIS). Für die Diagnosestellung war ausschließlich der behandelnde Arzt zuständig. Bei stationären Entlassungen wurden die Entlassdiagnosen durch administrative Kräfte nach vorheriger ärztlicher Feststellung dokumentiert. Grundlage für die Diagnosecodierung stellten ausschließlich klinische Untersuchungsergebnisse dar. Rettungsdienstliche Verdachtsdiagnosen oder eine initiale Triagierung bei Aufnahme in der Notaufnahme wurden explizit nicht berücksichtigt. Ausgelesen wurde jeweils die Behandlungsart (stationär oder ambulant), ICD-codierte Nebendiagnosen, Interventionen (d. h. Eingriffe am Herzen) und Blutwerte inklusive des bestimmten Troponinwerts. Dieser wird in der interdisziplinären Notaufnahme für jeden Patienten mit Aufnahmegrund „Synkope“ erhoben, unabhängig von thorakalen Beschwerden oder einer anderen zugehörigen Symptomatik. Für stationäre Patienten wurde zudem auch die entlassende Station und der Entlassungsgrund erfasst. Die einzelnen ICD-Codes der Nebendiagnosen wurden in übergeordneten Kategorien zusammengefasst (*Supplement: Diagnosen*). Nichtdokumentierte Diagnosen wurden als nichtvorhanden gewertet. Die Studie erfolgte mit Zustimmung der zuständigen Ethikkommission (Ethikkommission Klinikum rechts der Isar: 2023-507-S-KH), im Einklang mit nationalem Recht sowie gemäß der Deklaration von Helsinki von 1975 (in der aktuellen, überarbeiteten Fassung). Da es sich um eine retrospektive Studie handelt, war keine Einverständniserklärung der Studienteilnehmer erforderlich.

## Ergebnisse

**Abb. 1:**
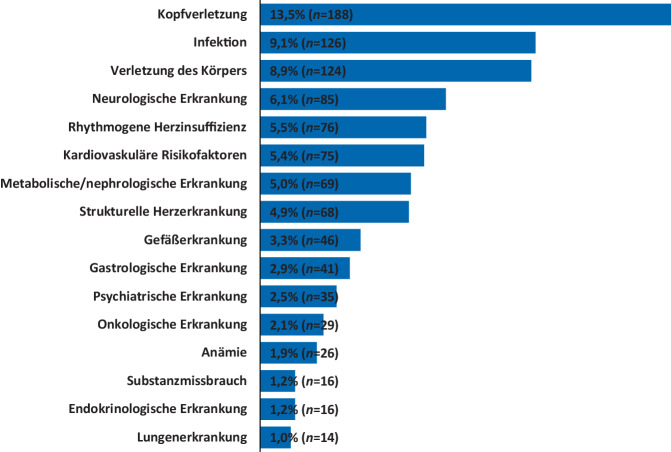
Inzidenz von Nebendiagnosen bei Notaufnahmepatienten mit Hauptdiagnose R55 „Synkope und Kollaps“. Patientengesamtzahl *n* = 1391 (siehe auch Tab. 1)

Im Zeitraum 2019 bis 2022 stellten sich insgesamt 1391 Patienten aus einem Gesamtkollektiv von 150.751 Notfallpatienten mit dem Leitsymptom Synkope in der interdisziplinären Notaufnahme vor. Von diesen waren 632 männlich (45,4 %) und 759 weiblich (54,6 %), das mittlere Alter betrug 53,1 Jahre (Tab. 1). Der Anteil an Patienten, die stationär versorgt wurden, lag bei 13,7 % (*n* = 190), die restlichen 1201 Patienten (86,3 %) konnten ambulant versorgt werden (Tab. 1). Die häufigsten Nebendiagnosen waren Kopfverletzung (13,5 %), Infektion (9,1 %), Verletzung des Körpers (8,9 %), neurologische Erkrankung (6,1 %), rhythmogene Herzinsuffizienz (5,5 %), kardiovaskuläre Risikofaktoren (5,4 %), metabolische/nephrologische Erkrankung (5,0 %) und strukturelle Herzerkrankung (4,9 %) (Abb. 1; Tab. 1). Bei 16 Patienten (1,2 %) erfolgte eine Herzkatheteruntersuchung, wobei 15 dieser Patienten (1,1 %) mindestens einen Stent erhielten. Zwölf Patienten (0,9 %) erhielten einen Herzschrittmacher, Defibrillator oder Ereignisrekorder.

**Tab. 1:** Patientencharakteristika und Nebendiagnosen

Patientencharakteristika	Stationär (*n* = 190)	Ambulant (*n* = 1201)	Insgesamt (*n* = 1391)
*Geschlecht*			
Männlich	96 (50,5 %)	536 (44,6 %)	632 (45,4 %)
Weiblich	94 (49,5 %)	665 (55,4 %)	759 (54,6 %)
*Alter*			
Mittleres Alter	67,4 Jahre	50,7 Jahre	53,1 Jahre
Standardabweichung	19,7 Jahre	22,6 Jahre	23,0 Jahre
Spanne	18–96 Jahre	16–97 Jahre	16–97 Jahre
*Nebendiagnosen*			
Rhythmogene Herzinsuffizienz	67 (35,3 %)	9 (0,7 %)	76 (5,5 %)
Strukturelle Herzerkrankung	57 (30 %)	11 (0,9 %)	68 (4,9 %)
Kardiovaskuläre Risikofaktoren	72 (37,9 %)	3 (0,2 %)	75 (5,4 %)
Lungenerkrankung	12 (6,3 %)	2 (0,2 %)	14 (1 %)
Kopfverletzung	48 (25,3 %)	140 (11,7 %)	188 (13,5 %)
Verletzung des Körpers	44 (23,2 %)	80 (6,7 %)	124 (8,9 %)
Substanzmissbrauch	12 (6,3 %)	4 (0,3 %)	16 (1,2 %)
Psychiatrische Erkrankung	20 (10,5 %)	15 (1,2 %)	35 (2,5 %)
Gefäßerkrankung	38 (20 %)	8 (0,7 %)	46 (3,3 %)
Infektion	110 (57,9 %)	16 (1,3 %)	126 (9,1 %)
Metabolisch/nephrologische Erkrankung	58 (30,5 %)	11 (0,9 %)	69 (5 %)
Neurologische Erkrankung	55 (28,9 %)	30 (2,5 %)	85 (6,1 %)
Onkologische Erkrankung	19 (10 %)	10 (0,8 %)	29 (2,1 %)
Gastroenterologische Erkrankungen	24 (12,6 %)	17 (1,4 %)	41 (2,9 %)
Endokrinologische Erkrankung	13 (6,8 %)	3 (0,2 %)	16 (1,2 %)
Anämie	21 (11,1 %)	5 (0,4 %)	26 (1,9 %)

**Abb. 2:**
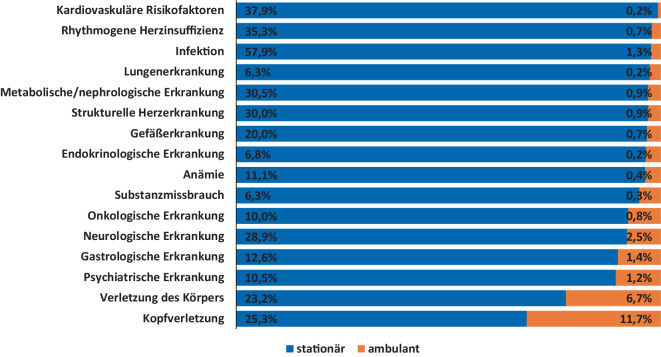
Inzidenz von Nebendiagnosen bei Notaufnahmepatienten mit Hauptdiagnose R55 „Synkope und Kollaps“ im Vergleich zwischen stationären und ambulanten Patienten. Die *Balken* zeigen das Verhältnis zwischen den relativen Häufigkeiten der jeweiligen Diagnose bei stationären und ambulanten Patienten (siehe auch Tab. 1)

**Abb. 3:**
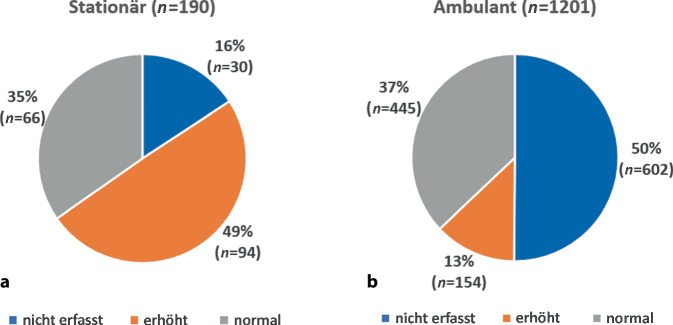
Troponin-T-Bestimmung bei Notaufnahmepatienten mit Hauptdiagnose R55 „Synkope und Kollaps“ im Vergleich zwischen stationären und ambulanten Patienten. **a** stationär **b** ambulant

**Abb. 4:**
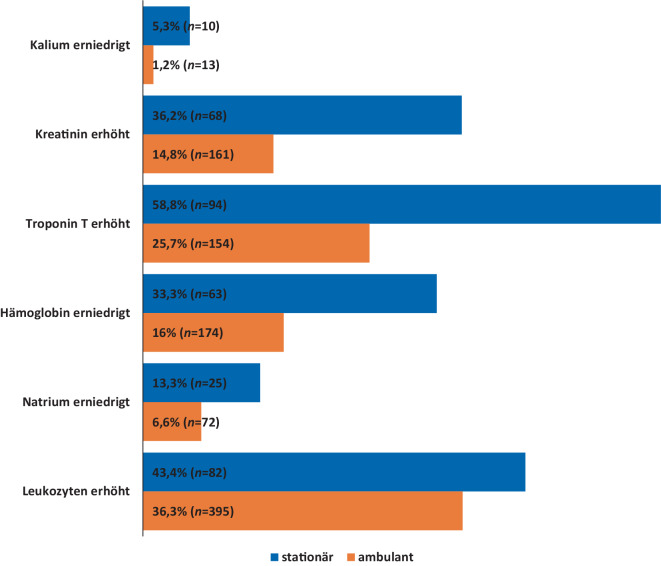
Relevante abnormale Blutwerte bei Notaufnahmepatienten mit Hauptdiagnose R55 „Synkope und Kollaps“ im Vergleich stationärer mit ambulanten Patienten. Die Anordnung folgt absteigend dem Maß der Überrepräsentation der pathologischen Blutwerte bei stationären Patienten. Die Prozentangaben beziehen sich auf alle Patienten der jeweiligen Gruppe, für die der betreffende Wert bestimmt wurde (siehe Tab. 2). Gruppengrößen: stationär (*n* = 190), ambulant (*n* = 1201)

Stationäre Patienten waren im Durchschnitt deutlich älter (67,4 vs. 50,7 Jahre) und wiesen ein nahezu ausgeglichenes Geschlechterverhältnis auf (Tab. 1). Die durchschnittliche Anzahl von Nebendiagnosen war bei stationären Patienten (3,5 Nebendiagnosen) deutlich höher als bei ambulanten Patienten (0,3 Nebendiagnosen). Sämtliche Nebendiagnosen lagen bei stationären Patienten mit einer deutlich höheren Inzidenz vor (Abb. 2; Tab. 1). So waren z. B. kardiovaskuläre Risikofaktoren (37,9 % vs. 0,2 %), rhythmogene Herzinsuffizienzen (35,3 % vs. 0,7 %), Infektionen (57,9 % vs. 1,3 %) und Lungenerkrankungen (6,3 % vs. 0,2 %) bei stationären Patienten deutlich häufiger (Abb. 2). Stationäre Patienten wiesen auch deutlich höhere Inzidenzen abnormaler Laborwerte auf (Tab. 2, Abb. 3 und 4). So war bei stationären Patienten z. B. die Inzidenz hoher Troponin-T-Werte, die auf eine Schädigung des Herzens z. B. wegen eines Herzinfarkts hinweisen, stark erhöht (Tab. 2; Abb. 3). Zudem waren bei stationären Patienten erhöhte Werte für Kreatinin (36,2 % vs. 14,8 %) und Leukozyten (43,4 % vs. 36,3 %) sowie erniedrigte Werte für Hämoglobin (33,3 % vs. 16,0 %), Kalium (5,3 % vs. 1,2 %) und Natrium (2,1 % vs. 0,6 %) deutlich häufiger (Tab. 2; Abb. 4). Von den stationären Patienten wurden 150 (78,9 %) primär internistisch, 25 (13,1 %) chirurgisch und 15 (0,8 %) anderen Fachbereichen zugeordnet aufgenommen. Nach erfolgter stationärer Behandlung konnten 172 (90,5 %) Patienten regulär entlassen werden, 8 (4,2 %) Fälle wurden an externe Krankenhäuser überwiesen, ein (0,5 %) Patient wurde in eine Rehabilitationseinrichtung überführt und bei 4 (2,1 %) Patienten musste im Lauf der Behandlung der Tod festgestellt werden. 5 (2,6 %) Patienten verließen gegen ärztlichen Rat die Klinik frühzeitig vor Ende der stationären Behandlung.

**Tab. 2:** Laborparameter bei stationären (*n* = 190) und ambulanten Patienten (*n* = 1201)

Laborparameter	Stationär (*n* = 190)	Ambulant (*n* = 1201)	Insgesamt (*n* = 1391)
*Troponin T*			
Erhöht	94 (58,8 %)	154 (25,7 %)	248 (32,7 %)
Normal	66 (41,2 %)	445 (74,3 %)	511 (67,3 %)
Ohne Angabe	30	602	632
*CK*			
Erhöht	44 (24,0 %)	255 (24,4 %)	299 (24,3 %)
Normal	139 (76,0 %)	792 (75,6 %)	931 (75,7 %)
Ohne Angabe	7	154	161
*CK-MB*			
Erhöht	19 (67,9 %)	40 (38,8 %)	59 (45,0 %)
Normal	9 (32,1 %)	63 (61,2 %)	72 (55,0 %)
Ohne Angabe	162	1098	1260
*Kalium*			
Erhöht	19 (10,1 %)	70 (6,4 %)	89 (7,0 %)
Erniedrigt	10 (5,3 %)	13 (1,2 %)	23 (1,8 %)
Normal	159 (84,6 %)	1005 (92,4 %)	1164 (91,2 %)
Ohne Angabe	2	113	115
*Natrium*			
Erhöht	4 (2,1 %)	7 (0,6 %)	11 (0,9 %)
Erniedrigt	25 (13,3 %)	72 (6,6 %)	97 (7,6 %)
Normal	159 (84,6 %)	1008 (92,7 %)	1167 (91,5 %)
Ohne Angabe	2	114	116
*Hämoglobin*			
Erhöht	7 (3,7 %)	73 (6,7 %)	80 (6,3 %)
Erniedrigt	63 (33,3 %)	174 (16,0 %)	237 (18,5 %)
Normal	119 (63,0 %)	842 (77,3 %)	961 (75,2 %)
Ohne Angabe	1	112	113
*HKT*			
Erhöht	16 (14,0 %)	112 (19,9 %)	128 (18,9 %)
Erniedrigt	58 (50,9 %)	139 (24,7 %)	197 (29,1 %)
Normal	40 (35,1 %)	312 (55,4 %)	352 (52,0 %)
Ohne Angabe	76	638	714
*Kreatinin*			
Erhöht	68 (36,2 %)	161 (14,8 %)	229 (17,9 %)
Normal	120 (63,8 %)	927 (85,2 %)	1047 (82,1 %)
Ohne Angabe	2	113	115
*GFR*			
Erniedrigt	135 (71,8 %)	520 (47,6 %)	655 (51,2 %)
Normal	53 (28,2 %)	572 (52,4 %)	625 (48,8 %)
Ohne Angabe	2	109	111
*Glukose*			
Erniedrigt	1 (0,7 %)	7 (0,7 %)	8 (0,7 %)
Normal	148 (99,3 %)	943 (99,3 %)	1091 (99,3 %)
Ohne Angabe	41	251	292
*Leukozyten*			
Erhöht	82 (43,4 %)	395 (36,3 %)	477 (37,3 %)
Erniedrigt	7 (3,7 %)	34 (3,1 %)	41 (3,2 %)
Normal	100 (52,9 %)	660 (60,6 %)	760 (59,5 %)
Ohne Angabe	1	112	113
*Thrombozyten*			
Erhöht	4 (2,2 %)	12 (1,1 %)	16 (1,3 %)
Erniedrigt	22 (11,8 %)	61 (5,6 %)	83 (6,6 %)
Normal	160 (86,0 %)	1008 (93,2 %)	1168 (92,2 %)
Ohne Angabe	4	120	124
*pH-Wert*			
Erhöht	16 (12,7 %)	35 (5,9 %)	51 (7,1 %)
Erniedrigt	44 (34,9 %)	215 (36,3 %)	259 (36,0 %)
Normal	66 (52,4 %)	343 (57,8 %)	409 (56,9 %)
Ohne Angabe	64	608	672
*INR*			
Erhöht	14 (7,5 %)	21 (2,0 %)	35 (2,8 %)
Normal	172 (92,5 %)	1046 (98,0 %)	1218 (97,2 %)
Ohne Angabe	4	134	138
*Quick-Wert*			
Erniedrigt	15 (9,1 %)	21 (2,3 %)	36 (3,3 %)
Normal	149 (90,9 %)	905 (97,7 %)	1054 (96,7 %)
Ohne Angabe	26	275	301
*aPTT*			
Erhöht	16 (8,6 %)	25 (2,4 %)	41 (3,3 %)
Erniedrigt	44 (23,7 %)	283 (27,1 %)	327 (26,6 %)
Normal	126 (67,7 %)	737 (70,5 %)	863 (70,1 %)
Ohne Angabe	4	156	160

## Diskussion

1–3 % aller Patienten der interdisziplinären Notaufnahmen in Deutschland stellen sich mit dem Leitsymptom „Synkope“ vor [7]. Die Komplexität des Krankheitsbilds mit diversen Differenzialdiagnosen und Genesen stellt eine klinische Herausforderung dar, derer sich unterschiedliche Leitlinien und Risikoassessments („risk stratification tools“) bereits angenommen haben [6, 8]. Fehldiagnosen sowie nicht vereinheitlichte klinische Prozesse führen zu hohen Kosten bei teils unnötig prolongierten Behandlungszeiträumen sowie vermeidbaren Hospitalisierungen. So liegen die mit Synkopebehandlungen assoziierten Kosten in den Vereinigten Staaten allein bei geschätzten 2,4 Mrd. US-Dollar [9]. Die Unkenntnis der Leitlinienempfehlungen und bereits erwähnter „Red-Flags“ (siehe CHESS-Kriterien) können teils gravierende Folgen für die Patienten haben [9]. In der Notaufnahme des Klinikums rechts der Isar der Technischen Universität München wird die initial beschriebene Leitlinie der ESC konsequent umgesetzt. Als Konsequenz wird ein Großteil der Patienten mit der Diagnose Synkope ambulant behandelt. In dieser Studie lag der Anteil bei 86,3 %. Nur 13,7 % der untersuchten Patienten mit einer Synkope mussten stationär aufgenommen werden. In der Literatur zeigt sich allerdings eine deutlich höhere Rate an stationären Aufnahmen bei Patienten mit Synkope [7]. So wird im Regelfall die Mehrheit aller Patienten mit Synkope (43–79,3 %) im Rahmen der Versorgung in Notaufnahmen stationär aufgenommen [10, 11]. Zur Absicherung bei fehlenden Pathologien trotz breiter Diagnostik wird fälschlicherweise eine stationäre Überwachung der Patienten mit Synkope angeordnet, auch wenn eine ambulante Therapie und direkte Entlassung medizinisch ausreichend wäre [8]. Die standardisierten ESC-Richtlinien werden somit oft nur unzureichend implementiert, sodass vermeidbare stationäre Aufnahmen erfolgen [12]. Auf Basis der erhobenen Werte dieser Studie lässt sich erkennen, dass die einheitliche Anwendung der empfohlenen ESC-Leitlinie in jedem Fall zu einer deutlichen Reduktion der stationären Aufnahmen der Patienten mit Synkope führen kann. So liegt die Hospitalisierungsrate von 13,7 % im Klinikum rechts der Isar der Technischen Universität München um bis zu 65,6 % unter den Angaben in der Literatur [11]. Die stationäre Aufnahme von Synkopepatienten erfolgt zumeist nur nach Vorliegen eines Risikokriteriums (siehe CHESS). Im direkten Vergleich korrelieren stationäre Aufnahmen deutlich mit abnormalen Laborwerten sowie dem Vorliegen von Nebendiagnosen wie von kardiovaskulären Vorerkrankungen. Diese Beobachtung stützt ebenfalls die Aussagen der Leitlinie [1].

In der untersuchten Kohorte lag die Mortalität stationärer Patienten bei 2,1 %. Generell scheint auch dies unterhalb der in der Literatur angegebenen Vergleichswerte zu liegen [13, 14], auch wenn Mortalität in vergangenen Studien meist in direktem Zusammenhang mit einer Nebendiagnose wie beispielsweise einer Lungenembolie analysiert wurde [15]. Ressourcen werden eingespart, ohne dass die Versorgungssicherheit der Patienten gefährdet wird.

Die Patientencharakteristika unserer Studie decken sich ebenfalls mit den Annahmen der Literatur zu Hochrisikofaktoren bei Patienten mit Synkope. Ein Alter > 60 sowie eine Herzvorerkrankung, ein unmittelbar mit dem Synkopegeschehen im Kontext stehendes Trauma oder auch begleitende Infektparameter scheinen hauptursächlich für ein schlechtes Outcome zu sein [16]. Stationäre Patienten mit Synkope in unserer Studie waren im Schnitt deutlich älter (67,4 vs. 50,4 Jahre), wiesen mehr Nebendiagnosen auf und zeigten häufig stark auffällige Blutlaborwerte und Infektparameter. Laut Leitlinie [1] stellt insbesondere eine kardiovaskuläre Synkope die mit gefährlichste Form der Synkope dar. Kardiovaskuläre Synkopen bedürfen laut Leitlinie in jedem Fall einer stationären Aufnahme und auch dies steht im Einklang mit den erhobenen Daten. So waren kardiovaskuläre Risikofaktoren im Verhältnis 37,9 % zu 0,2 % besonders bei stationären Patienten prävalent. Ferner fällt auf, dass sich fast 10 % aller Patienten mit Synkope auch mit einem manifestierbaren Infektgeschehen vorstellten. Infektionen sind bereits im Rahmen der COVID-19-Pandemie mit dem Auftreten von Synkopen assoziiert worden [17] und weitere Studien zur Prüfung einer Kausalität sowie Korrelation erscheinen sinnvoll. Während Traumata als Ursache für Synkopen bereits umfangreich erforscht sind, ist dies bei Infekten noch nicht der Fall [16].

Ein klares Verständnis der empfohlenen Risikostratifizierungsstrategien setzt die Erhebung und Zuordnung der passenden Patientencharakteristika voraus. Der Ansatz der vorliegenden Studie zeigt dies exemplarisch. Unter konsequenter Anwendung der ESC-Leitlinie kann die Hospitalisierungsrate von Patienten mit Synkope nachweislich verringert werden, sodass die Resourcenallokation optimiert sowie Kosten gespart werden [12]. Aufgrund mangelnder Studien zur aktuellen nationalen Strategie zum klinischen Umgang mit Patienten mit Synkope in Notfallaufnahmen sowie einer nur limitiert vorhandenen Studienlage zu eigentlichen Patientencharakteristika [7] versucht diese Studie mittels retrospektiv erhobener Daten, zu einer höheren Adhärenz zur entsprechenden Leitlinie zu animieren.

## Limitationen

Da es sich bei dieser Datenerhebung um eine retrospektive Analyse handelt, gelten alle bekannten Limitationen dieses Studiendesigns. Die erhobenen Daten beziehen sich ausschließlich auf die per Klassifikation erhobene Hauptdiagnose R55 und explizit nicht auf andere Primärdiagnosen, bei denen die Synkope als Sekundärsymptom auftreten kann. So erscheint auch die Interventionsrate mit Herzschrittmachern, Defibrillatoren oder Ereignisrekordern im Rahmen dieser Studie insgesamt niedrig, auch wenn sie die häufigste Intervention bei stationären Patienten mit Synkope darstellt [2]. Der ausschließliche Fokus auf die Synkope als Hauptdiagnose kann zu einer Verzerrung des Patientenkollektives führen. In dieser Studie wurden nur Notfallpatienten mit der Hauptdiagnose „Synkope“, die nicht über den Schockraum oder die Chest Pain Unit aufgenommen wurden, untersucht. Unsere Empfehlungen gelten somit ausschließlich für Patienten in einer Notaufnahme und nicht für solche in speziellen Notfalleinrichtungen wie einer Chest Pain Unit. Trotz sorgfältiger Diagnostik und Triagierung kann es ebenfalls zu Fehldiagnosen gekommen sein, sodass Patienten nach initial angenommenem epileptischem Anfall doch schlussendlich als Patienten mit Synkope weiterbehandelt wurden. Diese konnten in der vorliegenden Studie nicht inkludiert werden. Ferner lassen die Daten keine Rückschlüsse zu, ob Kopf- und Körperverletzungen infolge einer Synkope entstanden sind (Sturzverletzung) oder bereits schon Bestand hatten und möglicherweise ursächlich für die Synkope waren. Eine der mit Abstand häufigsten Nebendiagnosen bei stationären Patienten (Tab. 1) ist eine Infektion. Diese lässt sich mit der angeführten Datenlage ebenfalls nicht hinreichend differenzieren, sodass Rückschlüsse auf eventuelle Ursachen der Synkope schwierig zu rechtfertigen wären. Ob Infektionen primär ursächlich sind oder unabhängig als Symptom bei Synkopen auftreten, lässt sich nicht beantworten. Weitere Daten zur Patientenvorgeschichte und Anamnese bzw. eine umfangreichere Labordiagnostik, auch im Zeitverlauf, wären hier nützlich. Weitere Störvariablen der Studie können in der initialen Triagierung bzw. Codierung der Diagnosen oder auch in der Absenz eben jener Codierung zu finden sein. Patienten mit zugehörigen unvollständigen Datensätzen wurden aufgrund limitierter Vergleichbarkeit aus dieser Studie exkludiert. Schlussendlich sind auch keine Daten zu den Langzeitverläufen der Patienten vorhanden, die nach ambulanter Synkopebehandlung ohne stationäre Aufnahme direkt entlassen wurden. Dies stellt in der Tat eine Hauptlimitation unserer Studie dar, sodass getroffene Annahmen zumindest im Kontext einer fehlenden Krankheitsverlaufsperspektive kritisch evaluiert werden müssen. Etwaige ambulante Patienten, die entgegen der klinischen Entscheidung in der Notaufnahme in der Realität und im Verlauf doch von einer stationären Behandlung profitiert hätten, können so in den Datensätzen nicht berücksichtigt und eventuelle Verzerrungen der erhobenen Hospitalisierungsraten unzureichend dargestellt werden. Ambulant entlassene Patienten mit Hauptdiagnose Synkope hätten sich bei erneuter Symptomatik vorstellen und stationär aufgenommen werden können, wären jedoch dennoch im Rahmen dieser Studie als ambulante Fälle eingestuft worden. Es erscheint somit sinnvoll, eine prospektive Studie mit Fokus auf den Langzeitverlauf von Patienten mit Hautdiagnose Synkope anzuschließen, um die Ergebnisse dieser Studie weiter zu validieren. Auch wenn diese Limitation bestehen bleibt, ist zumindest von einem Trend hin zu fallenden Hospitalisierungsraten auszugehen, sofern eine Synkopediagnostik und -behandlung auf Basis der geltenden Leitlinien erfolgt.

## Fazit für die Praxis

- Das Krankheitsbild der Synkope in der Notfallversorgung ist multikausal und stellt somit eine ernste klinische Herausforderung in der Patientenversorgung dar.
- Uneinheitliche Umsetzungen der Leitlinien sowie zum Teil unvollständige Diagnostik führt zu unnötig vielen stationären Aufnahmen.
- Das bessere Verständnis der Patientencharakteristika von klinischen Fällen mit Synkope unter Einbezug der geltenden Leitlinientherapie führt zur signifikanten Reduktion der Hospitalisierungsraten. Auch eine standortspezifische SOP kann dadurch entwickelt werden, um eine Kontinuität der Diagnostik und Umsetzung der Leitlinie mit verbundenen Vorteilen sicherzustellen.
- Die Resourcenallokation in Notaufnahmen wird hierdurch optimiert, Kosten werden gespart und die allgemeine Patientenversorgung verbessert.

## Supplementary Information

Supplements Diagnosen

## Data Availability

Die erhobenen Datensätze können auf begründete Anfrage in anonymisierter Form beim korrespondierenden Autor angefordert werden. Die Daten befinden sich auf einem Datenspeicher am TUM-Universitätsklinikum, Klinikum rechts der Isar.
