# Supplementary material for: Characterization of patients with syncope in the emergency department—secondary diagnoses and laboratory parameters of inpatients versus outpatients
Source: Med Klin Intensivmed Notfmed. 2025 Feb 7;120(8):661–8. [Article in German] doi: 10.1007/s00063-024-01241-w (PMC12594738; doi:10.1007/s00063-024-01241-w)
Supplement: Supplementary file 1 — Supplements Diagnosen [file 63_2024_1241_MOESM1_ESM.docx]

**Supplements Diagnosen**

**Herzerkrankunegn**

- **Rhythmogene Herzinsuffizienz**

Z95 Vorhandensein von kardialen oder vaskulären Implantaten oder Transplantaten

I48 Vorhofflimmern und Vorhofflattern

I49 Sonstige kardiale Arrhythmien

R00 Störungen des Herzschlages

I45 Sonstige kardiale Erregungsleitungsstörungen

I44 Atrioventrikulärer Block und Linksschenkelblock

I47 Paroxysmale Tachykardie

Z96 Vorhandensein von anderen funktionellen Implantaten

Z45 Anpassung und Handhabung eines kardialen (elektronischen) Geräts

- **Strukturelle Herzerkrankung**

I20 Angina pectoris

I50 Nichtrheumatische Aortenklappenkrankheiten

I50 Herzinsuffizienz

I25 Chronische ischämische Herzkrankheit

I11 Hypertensive Herzkrankheit

I21 Akuter Myokardinfarkt

I27 Sonstige pulmonale Herzkrankheiten

I34 Nichtrheumatische Mitralklappenkrankheiten.

R60 Ödem, anderenorts nicht klassifiziert

I95 Hypotonie

I51 Komplikationen einer Herzkrankheit und ungenau beschriebene Herzkrankheit

R09 Sonstige Symptome, die das Kreislaufsystem und das Atmungssystem betreffen

I46 Herzstillstand

T82 Komplikationen durch Prothesen, Implantate oder Transplantate im Herzen und in den Gefäßen

I31 Perikarditis (chronisch) o.n.A.

R57 Schock, anderenorts nicht klassifiziert

I42 Kardiomyopathie

I05 Rheumatische Mitralklappenkrankheiten

I07 Rheumatische Trikuspidalklappenkrankheiten

Z99 Abhängigkeit (langzeitig) von unterstützenden Apparaten, medizinischen Geräten oder Hilfsmitteln, anderenorts nicht klassifiziert

I36 Nichtrheumatische Trikuspidalklappenkrankheiten

- **CVRF**

E78 Hypercholesterinämie

E11 Diabetes mellitus, Typ 2

I10 Essentielle (primäre) Hypertonie

**Lungenerkrankungen**

J44 Sonstige chronische obstruktive Lungenkrankheit

J96 Respiratorische Insuffizienz, anderenorts nicht klassifizier

J91 Pleuraerguss bei anderenorts klassifizierten Krankheiten

J69 Pneumonie durch feste und flüssige Substanzen

R06 Störungen der Atmung

J45 Asthma bronchiale

J98 Atemwegskrankheit (chronisch) o.n.A.

J90 Pleuraerguss, anderenorts nicht klassifiziert

J95 Krankheiten der Atemwege nach medizinischen Maßnahmen, anderenorts nicht klassifiziert

J84 Sonstige interstitielle Lungenkrankheiten

J82 Eosinophiles Lungeninfiltrat, anderenorts nicht klassifiziert

**Kopfverletzungen**

S01 Offene Wunde des Kopfes

S06 Gehirnerschütterung

S00 Oberflächliche Verletzung des Kopfes

S09 Sonstige und nicht näher bezeichnete Verletzungen des Kopfes

S02 Fraktur des Schädels und der Gesichtsschädelknochen

H11 Sonstige Affektionen der Konjunktiva

S05 Verletzung des Auges und der Orbita

I60 Subarachnoidalblutung

K14 Krankheiten der Zunge

T90 Folgen von Verletzungen des Kopfes.

**Verletzung des Körpers**

S22 Fraktur der Rippe(n), des Sternums und der Brustwirbelsäule.

S62 Fraktur im Bereich des Handgelenkes und der Hand

S61 Offene Wunde des Handgelenkes und der Hand

Z90 Verlust von Organen, anderenorts nicht klassifiziert

T84 Komplikationen durch orthopädische Endoprothesen, Implantate oder Transplantate

T14 Verletzung an einer nicht näher bezeichneten Körperregion

U50 Motorische Funktionseinschränkung

S40 Oberflächliche Verletzung der Schulter und des Oberarmes

S30 Oberflächliche Verletzung des Abdomens, der Lumbosakralgegend und des Beckens

S60 Oberflächliche Verletzung des Handgelenkes und der Hand Prellung eines oder mehrerer Finger o.n.A.

S20 Oberflächliche Verletzung des Thorax

S80 Oberflächliche Verletzung des Unterschenkels

T79 Bestimmte Frühkomplikationen eines Traumas, anderenorts nicht klassifiziert

L97 Ulcus cruris, anderenorts nicht klassifiziert

S50 Oberflächliche Verletzung des Unterarmes

S70 Oberflächliche Verletzung der Hüfte und des Oberschenkels

M51 Sonstige Bandscheibenschäden

S90 Oberflächliche Verletzung der Knöchelregion und des Fußes

Z43 Versorgung künstlicher Körperöffnungen

Z48 Andere Nachbehandlung nach chirurgischem Eingriff

S72 Fraktur des Femurs

T89 Sonstige näher bezeichnete Komplikationen eines Traumas

S32 Fraktur der Lendenwirbelsäule und des Beckens

S42 Fraktur im Bereich der Schulter und des Oberarmes

S71 Offene Wunde der Hüfte und des Oberschenkels o.n.A.

S12 Fraktur im Bereich des Halses Wirbel. Wirbelbogen

S93 Luxation, Verstauchung und Zerrung der Gelenke und Bänder in Höhe des oberen Sprunggelenkes und des Fußes

M24 Sonstige näher bezeichnete Gelenkschädigungen

S13 Luxation, Verstauchung und Zerrung von Gelenken und Bändern in Halshöhe

Z31 Fertilisationsfördernde Maßnahmen

Z98 Sonstige Zustände nach chirurgischem Eingriff

M54 Rückenschmerzen

S81 Offene Wunde des Unterschenkels o.n.A.

S03 Luxation, Verstauchung und Zerrung von Gelenken und Bändern des Kopfes.

M53 Sonstige Krankheiten der Wirbelsäule und des Rückens, anderenorts nicht klassifiziert

M23 Binnenschädigung des Kniegelenkes

T88 Sonstige Komplikationen bei chirurgischen Eingriffen und medizinischer Behandlung, anderenorts nicht klassifiziert

S16 Verletzung von Muskeln und Sehnen in Halshöhe

T81 Blutung und Hämatom als Komplikation eines Eingriffes, anderenorts nicht klassifiziert

S83 Luxation, Verstauchung und Zerrung des Kniegelenkes und von Bändern des Kniegelenkes ·

T09 Sonstige Verletzungen der Wirbelsäule und des Rumpfes, Höhe nicht näher bezeichnet.

M17 Gonarthrose [Arthrose des Kniegelenkes]

S86 Verletzung von Muskeln und Sehnen in Höhe des Unterschenkels

T00 Oberflächliche Verletzungen mit Beteiligung mehrerer Körperregionen

M35 Sonstige Krankheiten mit Systembeteiligung des Bindegewebes

S92 Fraktur des Fußes [ausgenommen oberes Sprunggelenk]

S63 Luxation, Verstauchung und Zerrung von Gelenken und Bändern in Höhe des Handgelenkes und der Hand

**Substanzmissbraauch**

T65 Toxische Wirkung sonstiger und nicht näher bezeichneter Substanzen

F05 Delir, nicht durch Alkohol oder andere psychotrope Substanzen bedingt

Y57 Unerwünschte Nebenwirkungen bei therapeutischer Anwendung von Arzneimitteln und Drogen

F19 Psychische und Verhaltensstörungen durch multiplen Substanzgebrauch und Konsum anderer psychotroper Substanzen

F14 Psychische und Verhaltensstörungen durch Kokain

**Psychiatrische Erkrankungen**

F33 Rezidivierende depressive Störung

F32 Depression

F10 Psychische und Verhaltensstörungen durch Alkohol

F06 Andere psychische Störungen aufgrund einer Schädigung oder Funktionsstörung des Gehirns oder einer körperlichen Krankheit

G47 Schlafstörungen

F43 Reaktionen auf schwere Belastungen und Anpassungsstörungen

F44 Dissoziative Störungen

F40 Phobische Störungen

F45 Somatisierungsstörung

F17 Psychische und Verhaltensstörungen durch Tabak

F13 Psychische und Verhaltensstörungen durch Sedativa oder Hypnotika

F41 Andere Angststörungen

F25 Schizoaffektive Störungen

F42 Zwangsstörung

F22 Wahnhafte Störung

**Gefäßerkrankungen**

I70 Atherosklerose ; Inkl.: Arteriolosklerose Arteriosklerose Arteriosklerotische Gefäßkrankheit

I72 Sonstiges Aneurysma und sonstige Dissektion

I71 Aortenaneurysma und -dissektion

I80 Thrombose, Phlebitis und Thrombophlebitis

I83 Varizen der unteren Extremitäten

I87 Venöse Insuffizienz (chronisch) (peripher), o.n.A.

I79 Krankheiten der Arterien, Arteriolen und Kapillaren bei anderenorts klassifizierten Krankheiten

I77 Sonstige Krankheiten der Arterien und Arteriolen

I83 Varizen der unteren Extremitäten

I65 Verschluss und Stenose präzerebraler Arterien ohne resultierenden Hirninfarkt

I67 Sonstige zerebrovaskuläre Krankheiten

**Infektionen**

Z11 Spezielle Verfahren zur Untersuchung auf infektiöse und parasitäre Krankheiten

U99 Spezielle Verfahren zur Untersuchung auf SARS-CoV-2

B02 Die Gürtelrose

Z22 Keimträger von Infektionskrankheiten. Inkl.: Verdachtsfälle

R22 Lokalisierte Schwellung, Raumforderung und Knoten der Haut und der Unterhaut

J10 Grippe

B96 Sonstige näher bezeichnete Bakterien als Ursache von Krankheiten, die in anderen Kapiteln klassifiziert sind

J15 Pneumonie durch Bakterien, anderenorts nicht klassifiziert

L03 Phlegmone: äußere männliche Genitalorgane

L30 Sonstige Dermatitis

J18 Pneumonie, Erreger nicht näher bezeichnet

R50 Fieber sonstiger und unbekannter Ursache

A49 Bakterielle Infektion nicht näher bezeichneter Lokalisation

B95 Streptokokken und Staphylokokken als Ursache von Krankheiten, die in anderen Kapiteln klassifiziert sind

R65 Systemisches inflammatorisches Response-Syndrom

U80 Grampositive Erreger mit bestimmten Antibiotikaresistenzen, die besondere therapeutische oder hygienische Maßnahmen erfordern

A41 Sonstige Sepsis

Z21 Asymptomatische HIV-Infektion

B98 Sonstige näher bezeichnete infektiöse Erreger als Ursache von Krankheiten, die in anderen Kapiteln klassifiziert sind

B18 Chronische Virushepatitis B ohne Delta-Virus: HBeAg positiv, ohne Entzündungsaktivität, hochreplikativ

J12 Viruspneumonie, anderenorts nicht klassifiziert

H70 Mastoiditis und verwandte Zustände

K75 Sonstige entzündliche Leberkrankheiten

L12 Pemphigoidkrankheiten

A46 Erysipel [Wundrose]

H10 Konjunktivitis

L89 Dekubitalgeschwür und Druckzone

J32 Sinusitis (chronisch) o.n.A.

A04 Sonstige bakterielle Darminfektionen

K61 Abszess in der Anal- und Rektalregion

K04 Pulpitis: akut. chronisch (hyperplastisch) (ulzerös) irreversibel

T80 Komplikationen nach Infusion, Transfusion oder Injektion zu therapeutischen Zwecken

B99 Sonstige und nicht näher bezeichnete Infektionskrankheiten

H92 Otalgie und Ohrenflus

**Metabolisch/Nephrologische Erkrankungen**

E87 Sonstige Störungen des Wasser- und Elektrolythaushaltes sowie des Säure-Basen-Gleichgewichts.

E79 Störungen des Purin- und Pyrimidinstoffwechsels

R63 Symptome, die die Nahrungs- und Flüssigkeitsaufnahme betreffen.

N39 Sonstige Krankheiten des Harnsystems

N13 Obstruktive Uropathie und Refluxuropathie

D41 Neubildung unsicheren oder unbekannten Verhaltens der Harnorgane.

N40 Prostatahyperplasie

N17 Akutes Nierenversagen

E86 Volumenmangel

N18 Chronische Nierenkrankheit

N28 Sonstige Krankheiten der Niere und des Ureters, anderenorts nicht klassifiziert

N36 Sonstige Krankheiten der Harnröhre

R64 Kachexie

D44 Neubildung unsicheren oder unbekannten Verhaltens der endokrinen Drüsen

N30 Zystitis

R39 Sonstige Symptome, die das Harnsystem betreffen

E66 Adipositas

E43 Nicht näher bezeichnete erhebliche Energie- und Eiweißmangelernährung

R56 Krämpfe, anderenorts nicht klassifiziert

N19 Nicht näher bezeichnete Niereninsuffizienz

**Neurologische Erkrankungen**

R29 Sonstige Symptome, die das Nervensystem und das Muskel-Skelett-System betreffen

R52 Schmerz, anderenorts nicht klassifiziert

G63 Polyneuropathie bei anderenorts klassifizierten Krankheiten.

R51 Kopfschmerz

R32 Nicht näher bezeichnete Harninkontinenz

R26 Störungen des Ganges und der Mobilität

R15 Stuhlinkontinenz

G20 Primäres Parkinson-Syndrom

R42 Schwindel und Taumel

R54 Senilität

F03 Nicht näher bezeichnete Demenz

R20 Sensibilitätsstörungen der Haut

G62 Sonstige Polyneuropathien

G82 Paraparese und Paraplegie, Tetraparese und Tetraplegie

G45 Zerebrale transitorische Ischämie und verwandte Syndrome

G93 Sonstige Krankheiten des Gehirns

R41 Sonstige Symptome, die das Erkennungsvermögen und das Bewusstsein betreffen

G25 Sonstige extrapyramidale Krankheiten und Bewegungsstörungen

G30 Alzheimer-Krankheit.

F00 Demenz bei Alzheimer-Krankheit

R07 Schmerzen in der vorderen Brustwand o.n.A.

F71 Mittelgradige Intelligenzminderung

H81 Störungen der Vestibularfunktion

U51 Kognitive Beeinträchtigung

G40 Epilepsie

R44 Sonstige Symptome, die die Sinneswahrnehmungen und das Wahrnehmungsvermögen betreffen

F20 Schizophrenie, schizotype und wahnhafte Störungen

R47 Sprech- und Sprachstörungen, anderenorts nicht klassifiziert.

H53 Sehstörungen

G91 Hydrozephalus

G43 Migräne

I69 Folgen einer zerebrovaskulären Krankheit

H91 Sonstiger Hörverlust

G81 Hemiparese und Hemiplegie

H46 Neuritis nervi optici

H47 Sonstige Affektionen des N. opticus

G83 Sonstige Lähmungssyndrome

R40 Somnolenz, Sopor und Koma

**Onkologische Erkrankungen**

C90 Plasmozytom und bösartige Plasmazellen-Neubildungen

C18 Bösartige Neubildung des Kolons.

D47 Sonstige Neubildungen unsicheren oder unbekannten Verhaltens des lymphatischen, blutbildenden und verwandten Gewebes

D61 Sonstige aplastische Anämien

C15 Bösartige Neubildung des Ösophagus

D32 Gutartige Neubildung der Meningen

C78 Sekundäre bösartige Neubildung der Atmungs- und Verdauungsorgane.

C34 Bösartige Neubildung der Bronchien und der Lunge.

C16 Bösartige Neubildung des Magens

C43 Bösartiges Melanom der Haut

C60 Bösartige Neubildung des Penis

C50 Bösartige Neubildung der Brustdrüse [Mamma]

C85 Sonstige und nicht näher bezeichnete Typen des Non-Hodgkin-Lymphoms

C82 Follikuläres Lymphom

J35 Chronische Krankheiten der Gaumenmandeln und der Rachenmandel

C32 Bösartige Neubildung des Larynx

D43 Neubildung unsicheren oder unbekannten Verhaltens des Gehirns und des Zentralnervensystems

C71 Bösartige Neubildung des Gehirns

C92 Myeloische Leukämie

C83 Nicht follikuläres Lymphom

C61 Bösartige Neubildung der Prostata

C77 Sekundäre und nicht näher bezeichnete bösartige Neubildung der Lymphknoten

C79 Sekundäre bösartige Neubildung an sonstigen und nicht näher bezeichneten Lokalisationen

C40 Bösartige Neubildung des Knochens und des Gelenkknorpels der Extremitäten

D70 Agranulozytose und Neutropenie

C44 Sonstige bösartige Neubildungen der Haut Bösartige Neubildung: Schweißdrüsen

C45 Mesotheliom

D25 Leiomyom des Uterus

D18 Hämangiom und Lymphangiom

I89 Sonstige nichtinfektiöse Krankheiten der Lymphgefäße und Lymphknoten

E85 Amyloidose

**Gastrologische Erkrankungen**

K44 Hernia diaphragmatica mit Einklemmung, ohne Gangrän

K29 Gastritis und Duodenitis

K40 Hernia inguinalis

R11 Übelkeit und Erbrechen

K25 Ulcus ventriculi

K21 Gastroösophageale Refluxkrankheit

R13 Dysphagie

K59 Sonstige funktionelle Darmstörungen

K12 Stomatitis und verwandte Krankheiten

K22 Sonstige Krankheiten des Ösophagus

R16 Hepatomegalie und Splenomegalie, anderenorts nicht klassifiziert

K26 Ulcus duodeni

K52 Sonstige nichtinfektiöse Gastroenteritis und Kolitis

A09 Sonstige und nicht näher bezeichnete Gastroenteritis und Kolitis nicht näher bezeichneten Ursprungs

K92 Sonstige Krankheiten des Verdauungssystems.

K71 idiosynkratische (unvorhersehbare) Leberkrankheit. toxische (vorhersehbare) Leberkrankheit.

I85 Ösophagusvarizen

K57 Divertikelkrankheit des Darmes

D13 Gutartige Neubildung sonstiger und ungenau bezeichneter Teile des Verdauungssystems

K85 Akute Pankreatitis

R18 Aszites

R10 Bauch- und Beckenschmerzen

**Endokrinologische Erkrankungen**

M81 Osteoporose ohne pathologische Fraktur

E03 Sonstige Hypothyreose

E05 Hyperthyreose [Thyreotoxikose]

E10 Diabetes mellitus, Typ 1

E06 Entzündung der Schilddrüse

R74 Abnorme Serumenzymwerte

**Anämie**

R58 Blutung, anderenorts nicht klassifiziert

D68 Von-Willebrand-Syndrom (VWS)

D64 Sonstige Anämien

D62 Akute Blutungsanämie

R04: Blutung aus den Atemwegen

D63 Anämie bei chronischen, anderenorts klassifizierten Krankheiten

R31 Nicht näher bezeichnete Hämaturie

I62 Sonstige nichttraumatische intrakranielle Blutung

N92 Zu starke, zu häufige oder unregelmäßige Menstruation

D69 Purpura und sonstige hämorrhagische Diathesen

D50 Eisenmangelanämie

**Nicht analysiert**

Z92 Medizinische Behandlung in der Eigenanamnese.

Z87 Andere Krankheiten oder Zustände in der Eigenanamnese

Z29 Notwendigkeit von anderen prophylaktischen Maßnahmen

X59 Akzidentelle Exposition gegenüber sonstigen und nicht näher bezeichneten Faktoren.

U07 Krankheiten mit unklarer Ätiologie, belegte und nicht belegte Schlüsselnummern

Z86 Bestimmte andere Krankheiten in der Eigenanamnese

Z74 Probleme mit Bezug auf Pflegebedürftigkeit

Z46 Versorgen mit und Anpassen von anderen medizinischen Geräten oder Hilfsmitteln

R53 Unwohlsein und Ermüdung

H69 Sonstige Krankheiten der Tuba auditiva

Z97 Vorhandensein anderer medizinischer Geräte oder Hilfsmittel

Z34 Überwachung einer normalen Schwangerschaft

H26 Sonstige Kataraktformen

Z13 Spezielle Verfahren zur Untersuchung auf sonstige Krankheiten oder Störungen

D72 Sonstige Krankheiten der Leukozyten

R49 Störungen der Stimme

H57 Sonstige Affektionen des Auges und der Augenanhangsgebilde

J38 Krankheiten der Stimmlippen und des Kehlkopfes, anderenorts nicht klassifiziert

H52 Akkommodationsstörungen und Refraktionsfehler

J17 Pneumonie bei anderenorts klassifizierten Krankheiten

H50 Sonstiger Strabismus

H58 Sonstige Affektionen des Auges und der Augenanhangsgebilde bei anderenorts klassifizierten Krankheiten.

O02 Sonstige abnorme Konzeptionsprodukte

O99 Sonstige Krankheiten der Mutter, die anderenorts klassifizierbar sind, die jedoch Schwangerschaft, Geburt und Wochenbett komplizieren

R46 Symptome, die das äußere Erscheinungsbild und das Verhalten betreffen

H40 Glaukom

O28 Abnorme Befunde bei der Screeninguntersuchung der Mutter zur pränatalen Diagnostik.

O36 Betreuung der Mutter wegen sonstiger festgestellter oder vermuteter Komplikationen beim Fetus.

U69 Sonstige sekundäre Schlüsselnummern für besondere Zwecke

Z51 Sonstige medizinische Behandlung

D37 Neubildung unsicheren oder unbekannten Verhaltens der Mundhöhle und der Verdauungsorgane

M62 Sonstige Muskelkrankheiten

O26 Betreuung der Mutter bei sonstigen Zuständen, die vorwiegend mit der Schwangerschaft verbunden sind.

K41 Hernia femoralis

O09 Schwangerschaftsdauer

N47 Vorhauthypertrophie, Phimose und Paraphimose

Q98 Sonstige Anomalien der Gonosomen bei männlichem Phänotyp, anderenorts nicht klassifiziert

J01 Akute Sinusitis

L70 Acne

K10 Sonstige Krankheiten der Kiefer

K07 Dentofaziale Anomalien [einschließlich fehlerhafter Okklusion]

O32 Betreuung der Mutter bei festgestellter oder vermuteter Lage- und Einstellungsanomalie des Fetus

K08 Sonstige Krankheiten der Zähne und des Zahnhalteapparates

J34 Sonstige Krankheiten der Nase und der Nasennebenhöhlen

H74 Sonstige Krankheiten des Mittelohres und des Warzenfortsatzes

L57 Hautveränderungen durch chronische Exposition gegenüber nichtionisierender Strahlung

Z32 Untersuchung und Test zur Feststellung einer Schwangerschaft
